# Three-Dimensional Skin Tissue Printing with Human Skin Cell Lines and Mouse Skin-Derived Epidermal and Dermal Cells

**DOI:** 10.4014/jmb.2111.11042

**Published:** 2021-12-15

**Authors:** Soojung Jin, You Na Oh, Yu Ri Son, Boguen Kwon, Jung-ha Park, Min jeong Gang, Byung Woo Kim, Hyun Ju Kwon

**Affiliations:** 1 Core-Facility Center for Tissue Regeneration, Dong-Eui University, Busan 47340, Republic of Korea; 2 Biopharmaceutical Engineering Major, Division of Applied Bioengineering, College of Engineering, Dong-Eui University, Busan 47340, Republic of Korea; 3 Blue-Bio Industry Regional Innovation Center, Dong-Eui University, Busan 47340, Republic of Korea

**Keywords:** 3D bioprinting, bioink, cytokeratin 14, keratinization, loricrin, skin

## Abstract

Since the skin covers most surfaces of the body, it is susceptible to damage, which can be fatal depending on the degree of injury to the skin because it defends against external attack and protects internal structures. Various types of artificial skin are being studied for transplantation to repair damaged skin, and recently, the production of replaceable skin using three-dimensional (3D) bioprinting technology has also been investigated. In this study, skin tissue was produced using a 3D bioprinter with human skin cell lines and cells extracted from mouse skin, and the printing conditions were optimized. Gelatin was used as a bioink, and fibrinogen and alginate were used for tissue hardening after printing. Printed skin tissue maintained a survival rate of 90% or more when cultured for 14 days. Culture conditions were established using 8 mM calcium chloride treatment and the skin tissue was exposed to air to optimize epidermal cell differentiation. The skin tissue was cultured for 14 days after differentiation induction by this optimized culture method, and immunofluorescent staining was performed using epidermal cell differentiation markers to investigate whether the epidermal cells had differentiated. After differentiation, loricrin, which is normally found in terminally differentiated epidermal cells, was observed in the cells at the tip of the epidermal layer, and cytokeratin 14 was expressed in the lower cells of the epidermis layer. Collectively, this study may provide optimized conditions for bioprinting and keratinization for three-dimensional skin production.

## Introduction

The skin is a large organ covering the surface of the body and protects the body from the external environment. The skin comprises the epidermis, dermis, and subcutaneous tissue [1, 2]. The epidermis, the outermost layer of the skin, is a self-renewing skin barrier that maintains homeostasis by repeating cell formation, differentiation, and breakout processes [3, 4]. Keratinocytes, which account for 95% of the epidermis, proliferate through cell division in the basal layer, and differentiate into keratinocytes as they move to the surface [5-7]. In addition, as the keratinocytes differentiate from the basal layer to the outermost layer of the epidermis, the stratum corneum, their shape becomes flatter [7]. The dermis is located between the epidermis and subcutaneous tissue and consists of dense connective tissue, which serves to maintain tension and elasticity in the skin [8,9]. The functions of the subcutaneous fat tissue, a fat layer several millimeters thick below the dermis, are body temperature control, shock absorption and energy storage [10-12].

Three-dimensional (3D) bioprinting is a technology used for constructing tissues or organs by stacking living cells in a desired shape or pattern [13]. There are three types of 3D bioprinting methods: inkjet, laser-assisted, and micro-extrusion. The inkjet printer is configured by replacing ink with a biomaterial and replacing paper with a moving stage [14]. Laser-assisted bioprinting is a method of making a structure by transferring energy to a material using a laser. Basically, it consists of a donor slide, an energy absorbing layer and a collector slide, and a structure is created by forming a bubble using a laser [15]. The micro-extrusion type of 3D bioprinting is the most commercially used, and there are two methods of injection: directly using pistons or screws, or indirectly using gas pressure. A micro-extrusion bioprinter generally consists of a temperature-controlled portion for biomaterial handling, a dispenser, and a stage. The dispenser and stage move on x, y and z axes to manufacture the product in stacked form. Multiple dispensers can be installed in the equipment to make tissues, organs and bones using various types of biomaterials such as gelatin, collagen, or extracellular matrix (ECM). Moreover, these materials can be used simultaneously without resetting the equipment [16, 17]. The dispenser of the micro-extrusion 3D bio printer forms a bead shape of the bioink and releases it to form a structure in a laminated manner [18].

Currently, numerous studies are actively underway around the world to produce artificial organs and artificial skin for customized medicine using 3D bioprinters. It has been reported that 3D cell printing has been investigated using tissue-specific ECM in vitro and in vivo [19, 20]. There are several reports that perfusable vascularized human skin equivalent has been developed and tested for skin graft onto immunodeficient mice [21, 22]. Research groups at the Institute of Regenerative Medicine at Wake Forest University have succeeded in transplanting ears printed with human cells into mice [23]. Researchers at Ohio State University have developed a technology that can 3D print organs or tissues inside the body using a bioink [24]. Additionally, there is a report that a customized 3D printing occlusion device has been developed for the production of a pig myocardial infarction model. This is expected to reduce the cost and increase the accuracy of the preclinical experimental stage of myocardial infarction [25]. In addition, Organovo in the United States has created and sold 3D liver tissues for drug toxicity testing, and successfully transplanted printed liver tissues to mice [26]. L'Oréal, a French cosmetics company, is working on a 3D printer to make artificial skins to test cosmetics and chemicals in response to animal testing regulations [27].

Until now, many safety and efficacy evaluations have been conducted using experimental animals to evaluate the impact of medicines, cosmetics, and chemicals on the human body. However, the need to develop and introduce alternative animal testing methods is being emphasized because of the increased ethical awareness of animal testing and the limitations of human application of animal testing results. Indeed, animal testing for safety evaluation in the cosmetics development process is prohibited in many countries including most of Europe. In place of animal experiments, various new forms of biotechnology and information technology, such as stem cell differentiation technology, tissue chip, organ chip, tissue reconstruction technology through 3D printing, and computer modeling, are being introduced to safety and effectiveness evaluation [28, 29].

The final goal of our research is to create customized artificial skin by directly extracting cells from human skin to replace animal testing. To this end, here, as a preceding study, we investigated the optimized conditions for the cell extraction from skin, the cell printing with higher cell survival rate, and the differentiation of keratinocytes using human- and mouse-derived cells.

## Materials and Methods

### Cell Culture of HaCaT and HFF-1

The HFF-1 cells (ATCC, USA) were incubated under conditions of 37°C, 5% CO_2_ using Dulbecco's Modified Eagle's Medium (DMEM; Welgene, Korea) containing 15% (v/v) fetal bovine serum (FBS; Hyclone, New Zealand) and 1% penicillin/streptomycin. The HaCaT cells (Addexbio, USA) were incubated under conditions of 37°C and 5% CO_2_ using DMEM containing 10% FBS and 1% penicillin/streptomycin.

### Cell Extraction from Mouse Skin

Eight-week-old C57BL/6J male mice (Central Laboratory of Animals; Korea) were maintained under specific pathogen-free conditions with a temperature of 22-24°C, humidity of 50-60%, and a lighting regimen of 12 h light and 12 h dark. All animal experiments were performed under an experimental protocol approved by the Ethics Review Committee for Animal Experimentation of Dong-eui University (A2018-001).

After sacrificing the mice, the skin was extracted and cut to about 5 × 5 mm, and then skin tissue was immersed in phosphate-buffered saline (PBS, pH 8.0) containing dispase (Sigma Aldrich, USA) solution (2.5 unit/ml) for O/N at 4°C. Dermis and epidermis were chopped finely for separation and treated with collagenase I and II (Sigma Aldrich) solution (2.5 unit/ml each) at 37°C for 3 h, followed by treatment with 0.05% trypsin at 37°C for 20 min. The reaction was stopped by adding the same amount of DMEM containing 20% FBS and antibiotics/antimycotics and filtering with a cell strainer (70 μm pore), followed by collection through centrifugation at 12,000 ×*g* for 20 min.

Extracted epidermal cells were incubated under 37°C and 5% CO_2_ using DMEM containing 20% FBS and antibiotics/antimycotics, and 1× human keratinocyte growth supplement (HKGS; GibcoBRL, USA). Extracted dermal cells were incubated at 37°C with 5% CO using DMEM containing 20% FBS and antibiotics/antimycotics.

### Production of the Bioink and 3D Bioprinting

Mouse epidermal cells or HaCaT cells (2 × 10^7^ cells/ml) and mouse dermal cells or HFF-1 cells (1 × 10^7^ cells/ml) were recovered to print 3D skin constructs. For the bioink preparation, cells were resolved in serum-free DMEM containing 20 mg/ml of fibrinogen (Sigma Aldrich), and then mixed with 10% gelatin (Sigma Aldrich) and 1%sodium alginate in a ratio of 1:2:1. For printing, the syringe was then filled with bioink, which contains cells, gelatin, fibrinogen, and alginate.

The skin construction was printed in a cylindrical shape with a diameter of 10 mm and a height of 3 mm on a 12-well transwell insert (pore size: 3 μm; Corning, USA) using a 3D bioprinter (Dr. Invivo; Rokit Healthcare, Korea). Transwell inserts provide an air-liquid interface environment in which epidermal cells are exposed to the air. As the printing condition parameters, a 22G syringe nozzle was used with 300 kPa pressure and filling density of 35%.

### Culture and Keratinization of 3D Printed Skin

Printed skin was cured in a DMEM containing 30% calcium chloride and 20 unit/ml thrombin (Sigma Aldrich) for 15 min. Thereafter, the skin construct was immersed in DMEM containing 15% FBS and 0.03 mM calcium ion to stabilize it for 2 days by LLI (liquid-liquid-interface) culture, and then ALI (air-liquid-interface) culture was performed for 14 days by adding DMEM containing 20% FBS, 8 mM calcium ion and 1× HKGS only to the bottom of the well for keratinization. The culture medium was exchanged once every two days.

### Fluorescence Labeling of Cells

For fluorescence labeling of cells, a CellTracker Fluorescent Probes Kit (Thermo Fisher Scientific, USA) was used and the experimental procedure followed the manufacturer’s instruction. HFF-1 cells (1 × 10^7^ cells/ml) and HaCaT cells (1 × 10^6^ cells/ml) were stained by Green CMFDA dye and Red CMPTX dye, respectively. Briefly, cells were resuspended in serum-free DMEM containing 25 μM of fluorescent dye for 30 min at 37°C.

### Measurement of Survival Rate through Live/Dead Staining

The 3D printed skin was cut thinly in cross section, placed on the slide glass, and washed with PBS, followed by incubation with 2 μM of calcein AM and ethidium homodimer-1 using the Live/Dead Viability/Cytotoxicity Kit (Thermo Fisher Scientific) for 30 min at room temperature. After staining with 4', 6-diamidino-2-phenylindole (DAPI; Sigma) for 10 min at room temperature, the skin construct was observed using a fluorescent microscope (Carl Zeiss, Germany) at the Core-Facility Center of Dong-eui University (Busan, Republic of Korea). For quantitative data, the fluorescence intensity of live cells was calculated at three independent points in the section, and calculated as a ratio to all cells.

### Preparation of Tissue Sections

For paraffin section preparation, the skin constructs and normal mouse skin were fixed with 4% formalin for O/N, dehydrated using the tissue processor (TP1020; Leica, USA), embedded in paraffin using the tissue embedding center (Tissue-Tek TEC5; Sakura Finetek, USA), and then sectioned to a thickness of 5 μm using the microtome (RM2245; Leica) at the Core-Facility Center of Dong-eui University (Busan, Republic of Korea).

For frozen section preparation, the printed skin constructs and normal mouse skin were fixed with 4% formalin for O/N, immersed in 10% sucrose/PBS for 1 h, and then immersed at 30% sucrose for O/N. Tissues were embedded in OCT compound and frozen in liquid nitrogen. Cryosectioning was performed to a thickness of 10 μm using a cryostat (CM1860; Leica) at -24°C.

### Hematoxilin-Eosin Staining

The paraffin sections were deparaffinized 2 times with xylene for 10 min and rehydrated, and then washed with distilled water. The slides were stained with hematoxylin for 8 min and soaked in acid alcohol for 30 sec, followed by dipping into ammonia solution for 30 sec. After washing in tap water, the slide samples were stained with eosin Y for 1 min. Slides were then dehydrated and soaked in xylene twice for 5 min, and coverslipped with a mounting medium for observation with an optical microscope (ECLIPSE Ci-L; Nikon, Japan).

### Immunofluorescence Staining

Frozen tissue sections were washed with PBS and non-specific binding signals were reduced with a blocking buffer containing 3% normal goat serum and 5% BSA for 1 h, followed by incubation with primary antibodies at 4°C for O/N. Mouse IgG or rabbit IgG (Sigma-Aldrich) was used as an isotype control. After washing with PBS, Alexa-488-conjugated anti-rabbit or Alexa-594-conjugated anti-mouse secondary antibodies were incubated for 1 h at room temperature. DAPI (1 μg/ml) was used for nuclear counterstaining and the slides were mounted with an anti-fade mounting buffer. The fluorescence signals were observed using a fluorescence microscope (Carl Zeiss), and photographed using the ZEN program (ZEISS). Primary antibodies against loricrin and cytokeratin 14 (CK14) were purchased from Abcam (UK), and secondary antibodies were purchased from Cell Signaling Technology (USA).

### Statistical Analysis

Data are presented as the mean ± SD from at least three independent experiments. Statistical comparisons between groups were performed by SPSS program followed by Student *t*-test. A value of *p* < 0.05 was considered statistically significant.

## Results

### Printing Conditions were Established to Maintain a High Survival Rate Using HaCaT and HFF-1 Cells

**Fig. 1:**
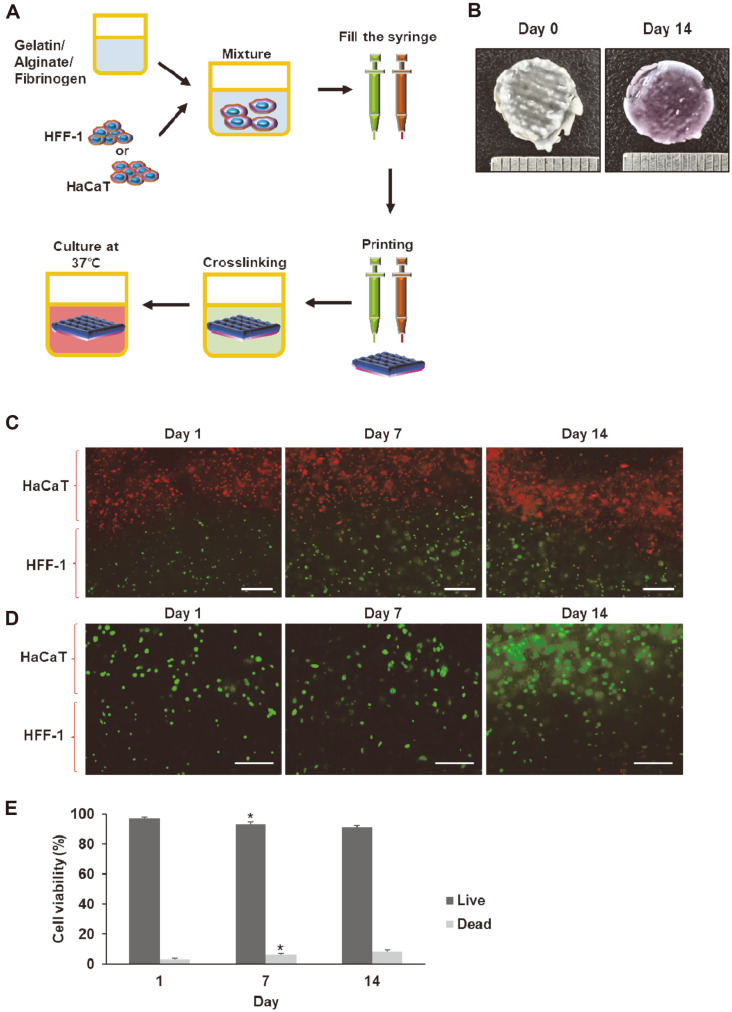
Printed and cultured 3D skin constructs using HaCaT and HFF-1 cells. (**A**) Schematic diagram depicting the bioink production, 3D skin printing and curing. (**B**) Photographs of printed skins before and after incubation. (**C**) HFF-1 and HaCaT cells were stained using Green CMFDA dye and Red CMPTX dye, respectively, followed by printing the cells. Scale bar, 200 μm. (**D**) Cell viability by fluorescent staining using Live/Dead Viability/Cytotoxicity Kit; live cells (green), dead cells (red). Scale bar, 200 μm. (**E**) Quantitative analysis of cell survival rate of printed skins by using the ZEN program (ZEISS). *p < 0.05 vs. fluorescence intensity at day 1.

To establish the conditions for artificial skin production using 3D bioprinting technology, first, skin constructs were printed using human foreskin fibroblasts (HFF-1) and human keratinocytes (HaCaT) by 3D bioprinter. They were printed according to the schematic diagram shown in Fig. 1A. The bioink was made by mixing gelatin, alginate, and fibrinogen with cells and printing was done in a cylinder style by micro-extrusion type 3D bioprinter. The constructs were cured using calcium ion and thrombin, followed by incubation for 14 days. As shown in Fig. 1B, we confirmed that the skin constructs printed with a diameter of 10 mm maintained their shape and did not shrink even after incubation for 14 days. To confirm whether the printed tissues maintained the stacked structure of the epidermal layer and the dermal layer, HaCaT cells were labeled with CMPTX dye (red) and HFF-1 cells were labeled with CMFDA dye (green). As shown in Fig. 1C, we observed that HFF-1 and HaCaT cells were not mixed together within the 3D structure and the stacked layers were well maintained. In addition, the proportion of live cells in the printed skin at 0, 7, and 14 days after incubation was relatively high at 97, 87, and 83%, respectively (Fig. 1D). These results showed that the bioink composition and printing conditions did not significantly affect cell viability.

### The Keratinization of Printed Skins Was Affected by the Concentration of Calcium Ion

**Fig. 2:**
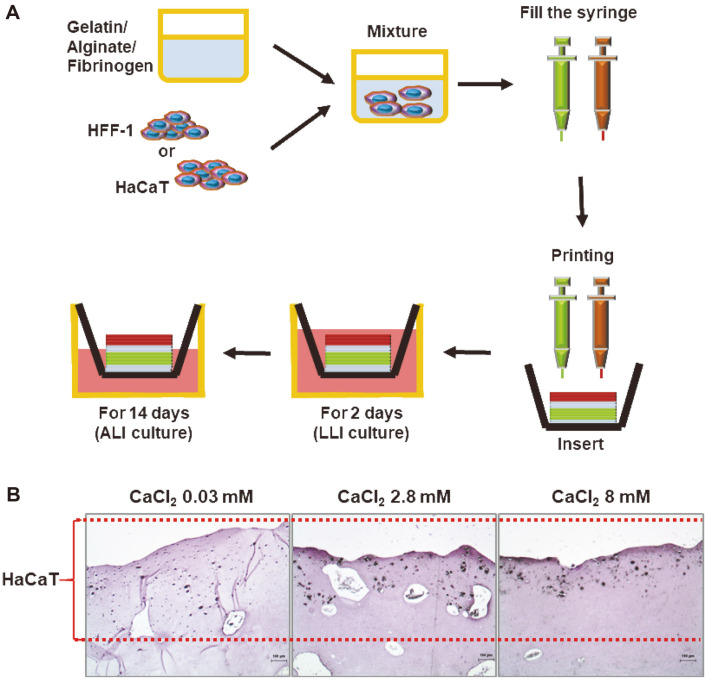
Culture conditions for keratinization of 3D printed skins using HaCaT and HFF-1 cells. (**A**) Schematic diagram showing skin printing and ALI culture; 3D printing using the bioink, LLI culture for 2 days (stabilization) and ALI culture for 14 days (keratinization). (**B**) Histological analysis by H&E staining. Printed skins were cultured under the different conditions of CaCl2 concentration (0.03, 2.8 or 8 mM). Scale bar, 100 μm.

To create printed tissues with similar characteristics to actual skin, we investigated the culture protocols to induce keratinization of the printed skin constructs. The culture method for inducing the keratinization is shown in Fig. 2A. The printed skins were stabilized for 2 days by immersion in media, called a liquid-liquid interface (LLI) culture, and then they were incubated without media in the upper side of the transwells, called an air-liquid interface (ALI) culture, for another 14 days (Fig. 2A). In addition, according to the report that calcium ion is important for the differentiation of HaCaT cells [30], the optimal conditions for keratinization by calcium ion concentration were tested. To obtain these conditions, calcium chloride was treated at a concentration (0.03, 2.8 or 8 mM) for 14 days during ALI culture period, and then the degree of keratinization of the printed skins was histologically observed. As shown in Fig. 2B, as the calcium chloride concentration increased, we confirmed that HaCaT cells in the epidermal layer were located close to the surface, indicating that calcium ion was one of the important inducers for keratinization in the epidermal layer of the skin constructs. As a result, for subsequent experiment we decided to cultivate the printed skins in a medium containing 8 mM of calcium chloride by ALI culture.

### The Printed Skins Using HaCaT and HFF-1 Cells Were Successfully Differentiated under the Optimized Conditions

**Fig. 3:**
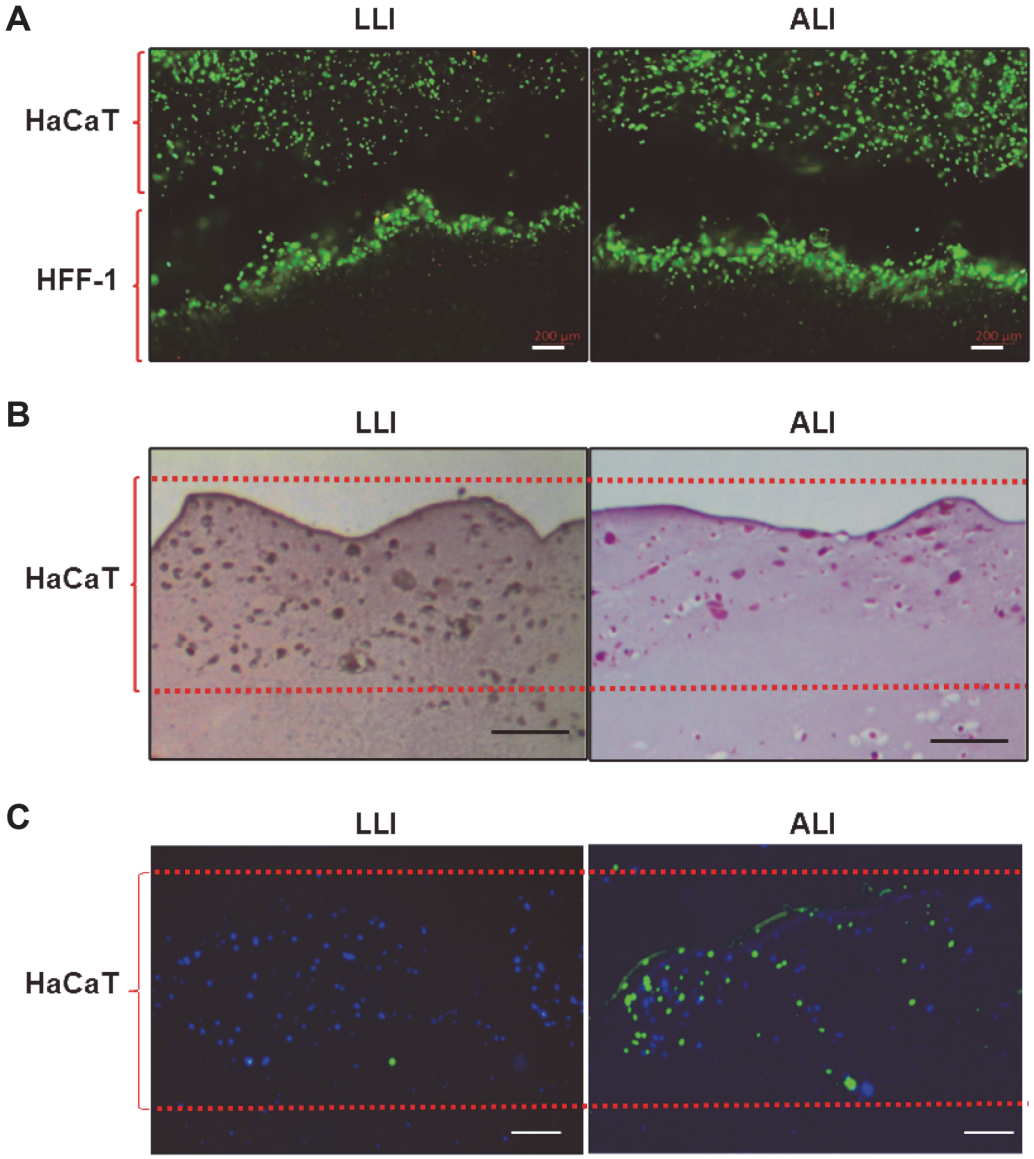
Histological studies of printed skins using HaCaT and HFF-1 cells after LLI and ALI culture. (**A**) Cell viability after LLI and ALI culture by fluorescent staining using Live/Dead Viability/Cytotoxicity Kit; live cells (green), dead cells (red). Scale bar, 200 μm. (**B**) H&E staining after LLI and ALI culture. Scale bar, 100 μm. (**C**) Immunofluorescent staining of cryosections of printed skins using keratinocyte differentiation marker protein, loricrin (green). DAPI was used as a nuclear counter stain. Scale bar, 200 μm.

To analyze the characteristics of printed skin tissues during keratinization, first, the cell viability of 3D printed skin before and after keratinization was examined. As shown in Fig. 3A, during keratinization, more living cells (green) were observed than dead cells (red) and maintained a high survival rate for 14 days of ALI culture. As a result of H&E staining on the paraffin sections of printed skins, HaCaT cells, which were evenly distributed before ALI culture, went up to the top of the epidermis as keratinization progressed (Fig. 3B). In addition, the expression of loricrin, one of the terminally differentiated stage markers of keratinocytes, increased with ALI culture and the addition of calcium ion, suggesting that the printed skin constructs were successfully stacked, survived and differentiated (Fig. 3C).

### Mouse Skin-Derived Cells Were Extracted and Printed to Construct the 3D Artificial Skin

**Fig. 4:**
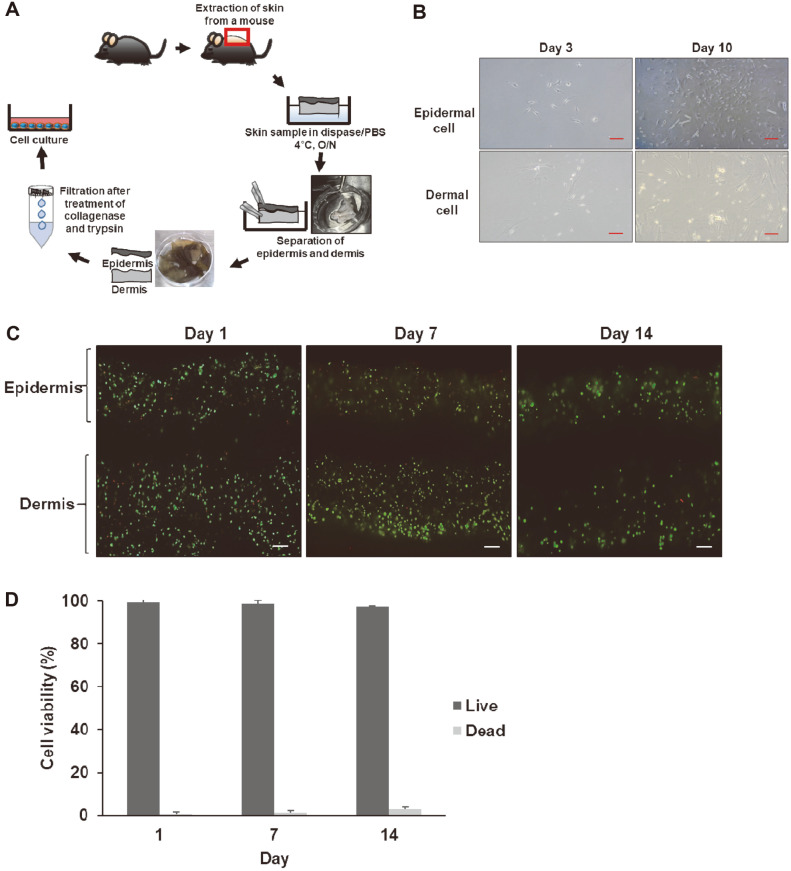
Extraction and 3D printing of primary cells from mouse skin. (**A**) The schematic diagram of primary cell extraction from mouse skin using various enzymes, such as dispase, collagenase, and trypsin. (**B**) The extracted mouse skin cells were cultured for 3 and 10 days. Scale bar, 100 μm. (**C**) Cell viability by fluorescent staining using Live/Dead Viability/ Cytotoxicity Kit; live cells (green), dead cells (red). Scale bar, 200 μm. (**D**) Quantitative analysis of cell survival rate of printed skins by using the ZEN program (ZEISS).

To establish the creating conditions for 3D printed skin tissue using primary cells extracted from actual biological skin, mouse epidermal and dermal cells were extracted, and then the cells were printed by 3D bioprinter based on the optimal printing and culture conditions determined using the human skin-derived cells thus far. Mouse skin-derived primary cell extraction was performed in the same manner as Fig. 4A. As shown in Fig. 4B, epidermal and dermal cells separated from the mouse skin were attached on the third day of culture and the number of cells increased over the incubation period. Skin constructs containing mouse epidermal and dermal cells were cured using calcium chloride and thrombin, and cultured with 8 mM calcium ions during ALI culture for 14 days. As shown in Fig. 4C, almost all cells in printed skins were observed to survive by Live/Dead staining on day 0, 7, and 14. In addition, from the results of quantitative analysis, it was confirmed that the proportion of living cells among existing cells in printed skins was 99%, 98%, and 97% on day 1, 7, and 14, respectively, after ALI culture (Fig. 4D). These results showed that optimal conditions with a high cell viability were established when skin constructs were printed and cultured using epidermal and dermal cells extracted from mouse skin tissue.

### The Skin Constructs Using Mouse Epidermal and Dermal Cells Showed the Upregulation of Differentiation Markers for Keratinocytes

**Fig. 5:**
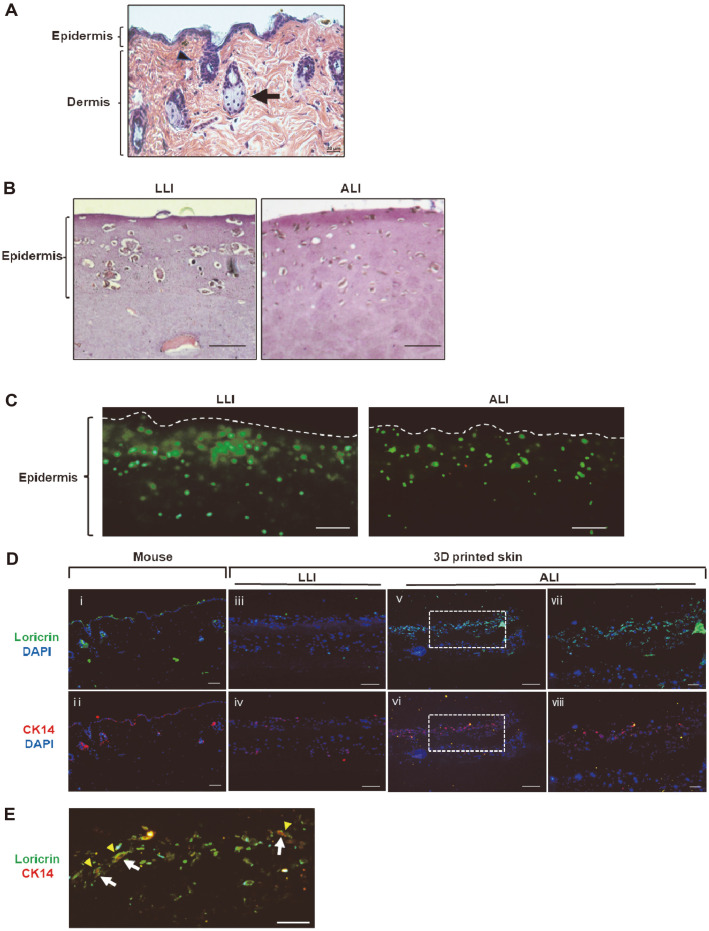
Histological studies of printed skins using mouse epidermal and dermal cells after LLI and ALI culture. (**A**) H&E staining of normal mouse skin section. Arrowhead; hair follicle, Arrow; sebaceous gland. Scale bar, 20 μm. (**B**) H&E staining of printed skins after LLI and ALI culture. Scale bar, 100 μm. (**C**) Cell viability by fluorescent staining using Live/Dead Viability/Cytotoxicity Kit; live cells (green), dead cells (red). Scale bar, 200 μm. (**D**) Immunofluorescent staining of cryosections of normal mouse skin and printed skins using keratinocyte differentiation marker proteins, loricrin (green) and CK14 (red). DAPI was used as a nuclear counter stain. (i, ii) Normal mouse epidermis. Scale bar, 50 μm. (iii, iv) 3D printed skin by LLI culture. Scale bar, 200 μm. (v, vi) 3D printed skin by ALI culture. Scale bar, 200 μm. (vii, viii) Inset images of v and vi. Scale bar, 100 μm. (**E**) Merged image of vii and viii. Arrowhead; loricrin expression in the upper cells. Arrow; CK14 expression below in the loricrin-positive cells. Scale bar, 100 μm.

Histological analysis was performed to investigate the biosimilarity between the printed artificial skins and mouse normal skin tissues. As shown in Fig. 5A, in normal mouse skin tissues, epidermis, which was made in the keratinized layers, was in the upper part of the skin, and hair follicle and sebaceous gland could be identified in the dermis. Comparative observation of the printed skins showed that epidermal cells were evenly located in the epidermal layer during LLI culture, whereas cells moved up to the tip of the epidermal layer during ALI culture with 8 mM calcium ion (Fig. 5B). After ALI incubation for 14 days, most cells (green) were alive, and cells were distributed on the upper side of the epidermal layer, whereas cells were widely distributed in the epidermal layer during LLI culture (Fig. 5C).

To confirm whether the differentiation of artificial skin is similar to that of mouse skin, the expression patterns of keratinization markers were examined by immunofluorescent staining using tissue sections of normal mouse skin and printed skin. Loricrin and CK14, used in this experiment, are differentiation indicators of keratinization, and the expression site and time of these markers within the epidermis layer are different during keratinization [31, 32]. As shown in Fig. 5D, in normal mouse skin tissues (i, ii), loricrin was expressed in terminally differentiated epidermal cells and CK14 was expressed in cells located in the basal layer. In the case of printed skins, CK14 was weakly expressed and loricrin was hardly expressed in epidermal cells during LLI culture for 2 days after printing (Fig. 5D. iii, iv). On the other hand, when ALI culture was performed for 14 days after LLI culture, the expression of loricrin and CK14 was increased (Fig. 5D. v, vi), and loricrin and CK14 were expressed especially in the upper cells of the epidermis layer and below in the loricrin-positive cells, respectively (Fig. 5D. vii, viii and Fig. 5E). Taken together, when the printed skin constructs were cultured using ALI culture method in a medium containing 8 mM calcium ion, the keratinization of the epidermal cells was well achieved, indicating that the artificial skins printed with mouse skin-derived primary cells were differentiated similar to the actual mouse skin epidermis.

## Discussion

The skin is an organ that performs the vital function of protecting the human body from the external environment and harmful pathogens. So far, medical replacement methods, including autograft (replacement with the patient's own skin), allograft (replacement with donor skin), and xenograft (replacement with heterogeneous skin), have been widely used as treatments for skin damaged due to diseases or accidents. However, these methods are limited to very small-scale skin replacements, so alternative therapies are still required to step over these thresholds [33, 34]. As an alternative method, various mechanical and biological approaches have recently been attempted to produce 3D skin tissue. Among them, 3D bioprinting technology with various customized cells, biomaterials, and mechanical properties is emerging as a very promising technology [35]. In addition, such replaceable artificial skin is being actively studied not only for skin regeneration but also for drug screening, functional evaluation and disease model production [36].

In this study, we investigated the conditions of skin tissue printing using gelatin as a bioink for the screening and functional evaluation of cosmetic materials. Human skin-derived cell lines, HaCaT and HFF-1, were used to determine the appropriate number of cells, bioink composition, size of the printed skin, curing conditions, and keratinocyte differentiation conditions. To serve as the foundation for construction of customized artificial skin, primary epidermal cells and primary dermal cells extracted from the skin of mice were used for 3D bioprinting and for keratinization, followed by the examination of the survival rate and the degree of differentiation using molecular markers, such as CK14 and loricrin.

The epidermis, which primarily consists of keratinocytes, is located at the outermost of the three layers that make up the skin, and is composed of multiple layers: stratum corneum, stratum granulosum, spinous cell layer, and basal layer, in descending order [37, 38]. As differentiation progresses, keratinocytes in the epidermis become flattened from the basal layer to the outer layer and finally the stratum corneum is formed. These processes are called keratinization, a process of forming an epidermal barrier that results in producing the various types of cytokeratins [39]. Cytokeratins are a component of intermediate filaments found in intracytoplasmic cytoskeleton of epithelial cells and help cells withstand mechanical stress [40]. There are two categories of cytokeratins: acid type I and basic type II cytokeratins, and 20 types of cytokeratin have been found to date. The expression of each cytokeratin subset depends on the type of epithelial cell and the differentiation stage of epithelial cells [41]. In particular, cytokeratin 14 (CK14) is mainly produced by keratinocytes distributed in the basal layer and cytokeratin 10 (CK10) is mainly expressed in the stratum corneum [42]. In addition, loricrin, one of the components of the conformed cell envelope, is used as a molecular marker for terminally differentiated epidermal cells during the keratinization [43]. It has been reported that calcium plays an important role in the differentiation of keratinocytes, which is controlled by the calcium concentration gradient [38, 44, 45]. In addition, in the case of terminally differentiated cells present at the uppermost layer of the skin, contact with air is one of the important requirements for forming a well-developed and differentiated epidermis [46].

Therefore, in this experiment, the concentration gradient of calcium ion was formed in the 3D printed skins, and it is believed that these microenvironments enabled keratinization of mouse epidermal cells. The differentiation of the epidermal cells in printed skins under these culture conditions occurred, and it was observed that the epidermal cells moved toward the top of the epidermis layer. However, the differentiated keratinocytes were scattered rather than forming a binding monolayer. To solve this problem, future studies intend to complete keratinization through various methods, such as extending the culture period or observing the keratinization process that rises to the surface by thinly covering the gelatin on top of it after printing the epidermis. In addition, the molecular marker loricrin was rarely expressed during LLI culture, whereas it was mainly expressed in the cells localized on the upper side of the epidermal layer after 14 days of ALI culture. On the other hand, the expression of CK14 was widely observed in the epidermal layer during LLI cultivation period, but after ALI culture, CK14 was mainly expressed in the basal cells of the upper epidermal layer. After ALI culture, the expression of loricrin was increased and the expression of CK14 was mainly expressed in cells located in the lower layer of the loricrin-positive cells, raising the possibility that keratinization of epidermal cells was progressing properly. However, the loricrin expression was also observed in cells located below the end of the epidermis. These results may be due to the difference in the concentration gradient of calcium ions within the printed skins, and future studies will be conducted to confirm this.

In conclusion, 3D bioprinting was performed using the gelatin-based bioink containing epidermal and dermal cells extracted from mouse skin, and then printed skin constructs were successfully cultured and differentiated in ALI microenvironment under the calcium ion gradient. This study suggests that these optimal printing and culture methods can be used for producing customized human skin using extracted skin cells.
